# From single-molecule spectroscopy to super-resolution imaging of the neuron: a review

**DOI:** 10.1088/2050-6120/4/2/022004

**Published:** 2016-06-24

**Authors:** Romain F Laine, Gabriele S Kaminski Schierle, Sebastian van de Linde, Clemens F Kaminski

**Affiliations:** 1 Laser Analytics Group, Department of Chemical Engineering and Biotechnology, Cambridge University, Pembroke Street, Cambridge, CB2 3RA, UK; 2 Department of Biotechnology and Biophysics, Julius-Maximilians-University, Am Hubland, D-97074 Würzburg, Germany; vdlinde@uni-wuerzburg.de; cfk23@cam.ac.uk

**Keywords:** single-molecule localization microscopy, neurodegeneration, neuroscience, bioimaging, super-resolution microscopy, protein misfolding and aggregation

## Abstract

For more than 20 years, single-molecule spectroscopy has been providing invaluable insights into nature at the molecular level. The field has received a powerful boost with the development of the technique into super-resolution imaging methods, ca. 10 years ago, which overcome the limitations imposed by optical diffraction. Today, single molecule super-resolution imaging is routinely used in the study of macromolecular function and structure in the cell. Concomitantly, computational methods have been developed that provide information on numbers and positions of molecules at the nanometer-scale. In this overview, we outline the technical developments that have led to the emergence of localization microscopy techniques from single-molecule spectroscopy. We then provide a comprehensive review on the application of the technique in the field of neuroscience research.

## From single-molecule spectroscopy to super-resolution imaging

Fluorescence microscopy permits cellular processes to be imaged with high specificity and with a lateral resolution of around 200 nm [1]. However, numerous cellular structures, such as vesicles or molecular complexes, remain inaccessible for imaging with conventional fluorescence techniques. State-of-the-art super-resolution imaging methods, on the other hand, can now routinely improve on the ~200 nm resolution limit by a factor of ten and more, and thus have opened the door for the study of finer cellular ultrastructure, such as the cytoskeletal organization in axons [2]. The conceptual achievement and future potential of super-resolution imaging techniques were acknowledged with the award of the 2014 Nobel Prize in Chemistry to Eric Betzig and W E Moerner for single-molecule-based super-resolution microscopy (or SMLM for single-molecule localization microcopy) and to Stefan Hell for stimulated emission depletion (STED) microscopy [3]. Structured illumination microscopy, SIM, pioneered by the late Mats Gustafsson, is another super-resolution method that is rapidly gaining in popularity [4–7]. All these approaches enable the diffraction limitations in conventional light microscopy to be overcome and have their advantages under particular experimental conditions.

**Figure 1.**
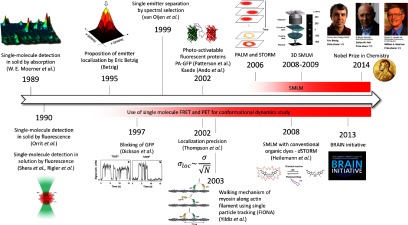
Theoretical and technical developments in single molecule spectroscopy: from birth (ca. 1989) to Nobel Prize to Eric Betzig, W E Moerner and Stefan Hell in 2014. Single-molecule spectroscopy contributed to the emergence of super-resolution microscopy, but also to the development of conformational study at the single molecule level. The potentials of super-resolution for the study of neuroscience was in particular highlighted by the launch of the BRAIN initiative. Modified with permission from [12, 30, 42, 80]. Copyright 1999 AAAS, 2004 Macmillan, 1997 Macmillan and 2008 AAAS, respectively.

Many technical innovations have taken place over the last decade and today super-resolution methods are widely available, even as commercial solutions, and their use, even by non-specialists, is becoming routine. Methods based on single-molecule imaging are becoming particularly widespread since they are technically simple to implement yet typically achieve a resolution in the 10–20 nm range. In this review, we focus the storyline on how progress in single-molecule spectroscopy has led to the advent of advanced localization microscopies and how these methods are now transforming biomedical research (figure 1). The neurosciences, and, in particular, research into so-called protein misfolding diseases have benefitted greatly from these recent developments and we use examples from the recent literature to illustrate how SMLM is shedding light on the molecular anatomy of the neuron, offering ground breaking information on its structural and functional components and insights into the molecular mechanisms behind devastating diseases such as Alzheimer’s and Parkinson’s.

The first individual molecule was detected by Moerner and Kador in 1989 [8]. For this pioneering work, para-terphenyl crystals were doped with pentacence and the absorption spectrum of single molecules was measured at liquid helium temperature. A year later, the experiment was repeated by Orrit and Bernard who used fluorescence detection instead of absorption [9]. In the latter study, pentacene was excited with a pulsed dye laser and fluorescence detected with a photomultiplier. The same year, Rigler *et al* and Shera *et al* succeeded in detecting the fluorescence of single molecules in solution [10, 11]. Shera *et al* focused the beam of a pulsed Nd:YAG laser into a flow cell containing a solution with 100 fM of Rhodamine 6G and detected the fluorescence with a multi-channel plate as single-molecules diffused through the laser focus. These were extraordinary achievements with the technology available at the time, but owing to dramatic advances in laser and detector technologies in intervening years, single molecule detection is today routinely achieved in laboratories around the world, with compact, cost effective technologies, such as diode lasers and CCD cameras.

The power of single-molecule methods was recognized from the start, and led to numerous applications, most especially in the biochemical sciences [12–14]. For instance, single-molecule fluorescence methods were used to unravel enzymatic reactions at the single-molecule level. An early example is the study of cholesterol oxidase activity in real time, exploiting the fact that the enzyme’s active site is fluorescent in its oxidized form, and non-fluorescent in its reduced form [15]. In another impressive experiment, Noji *et al* were able to observe directly the rotary action of F1-ATPase by attaching the *γ*-subunit to a fluorescently labelled actin filament [16]. Single-molecule fluorescent spectroscopy is widely exploited to detect conformational changes in macromolecular protein complexes. An example is the synaptotagmin 1–SNARE fusion complex, crucial for neurotransmitter release: Choi *et al* combined single molecule detection with Förster resonance energy transfer (FRET) to monitor conformational changes in the synaptotagmin C2 domains upon SNARE binding, and were able to deduce structural models with an experimental resolution of between 2 and 10 nm [17]. Similarly, photoinduced electron transfer (PET) spectroscopy is capable of detecting the dynamics of molecules at a resolution of ~1 nm [18].

These are examples of spectroscopic methods coupled to single molecule detection, which indirectly offer resolution far below the classical limit imposed by optical diffraction [19, 20]. Although such methods do not improve resolution of microscopic images *per se*, they are nevertheless conceptual forerunners of single-molecule super-resolution imaging methods, in exploiting a photophysical or photochemical process that takes place over spatial scales much smaller than the wavelength of light itself to offer information on the nanometer scale. Although single-molecule assays are not restricted to single-molecule fluorescence, we will here focus on their applications that led to the birth of SMLM.

The precise localization of single molecule emitters with resolution and noise limited instruments, e.g. with two-dimensional (2D) detector arrays, was already demonstrated both theoretically [21] and experimentally [22] upon the realization that diffraction does not *per se* pose a limitation on one’s ability to locate an individual fluorophore with good precision. A diffraction limited image of a single fluorophore, yields a photon emission pattern on the detector that is distributed according to the point spread function, PSF, of the microscope. The latter can be modeled and fitted with a spatial photon distribution function, typically using a Gaussian function for computational convenience, whose centroid gives a more precise estimation of the fluorophore position than imposed by the ~200 nm diffraction limit [23]. In fact, the fidelity with which the centroid position can be estimated is governed by the size of the PSF, the signal to noise ratio, and the number of photons that are detected. The precision of the localization, typically described as the standard deviation of the localization estimator, scales as the inverse of the square root of the photon number [24–27]. Also, in addition to sufficient fluorescence signal, the only other criterion required is that the density of imaged fluorophores is sparse, so that individual PSFs do not exhibit a significant degree of overlap in individual images. This principle was already exploited in early single molecule tracking experiments [22].

The use of organic dyes and quantum dots, with their high fluorescence quantum yield, permits very high localization precisions to be achieved, down to the single-digit nanometer range. For instance, early single-molecule tracking experiments, referred to as fluorescence imaging with one nanometer accuracy (FIONA) [28] revealed the mechanism that permits myosin to walk along actin filaments [29, 30]. More recently, postsynaptic receptor proteins in living neurons were labeled with small quantum dots and their diffusion behavior determined within the synaptic cleft, using similar concepts [31]. In another study, single molecule tracking revealed the reversible formation of functional hydrogels from protein linked to motoneuron disease [32].

However, single-particle (or single-molecule) tracking techniques require that only a single emitter is present within a diffraction-limited region (DLR). Therefore, the emitter localization approach cannot be immediately extended to obtain super-resolved images, because in densely labeled structures the distances between fluorophores are smaller than the DLR and consequently their emission patterns overlap and lead to image blur.

In 1995, Betzig proposed that fluorescence imaging beyond the diffraction barrier might be possible if the individual emitters (molecules) within the DLR have unique optical characteristics [33] such that they become optically discernable. He thus proposed that molecules must be ‘identified and isolated through one or more distinguishing optical characteristics’. The spatial coordinates of each molecule can then be determined and, finally, ‘the complete set of coordinates for all features can then be used to reconstruct the final image in which the relative positions of the features are shown’ [33]. This concept was originally proposed to be combined with near-field scanning optical microscopy at cryogenic temperatures, but it embodies completely the principle of the ‘localization technique’ that is now widely in use to achieve optical super-resolution with conventional wide-field microscopes operating at room temperature.

In the work of van Oijen *et al* in 1999, a technique had already been proposed for far-field super-resolution microscopy on the principle that suitable molecules can be spectrally distinguished with high-resolution laser spectroscopy [34]. The work was based on a 1985 publication by Burns *et al* which demonstrated the spatial discrimination of two molecules separated by a distance below the diffraction limit through exploitation of different spectral characteristics of certain dyes [35]. Similar attempts to discriminate multiple emitters within the DLR were made using differences in emission spectra [36, 37] or fluorescence lifetimes [38]. At the turn of this century, the separation of individual fluorophores was achieved through photobleaching [39] or temporal intensity fluctuations by exploiting the blinking properties of quantum dots (QDs) [40].

However, all of the approaches described above required a low number of molecules to be present within a DLR, and thus the reconstruction of densely labeled and complex biological structures remained challenging.

A milestone was reached with the advent of photoswitchable fluorophores. These are molecules whose fluorescence can be controlled by external means, such as photoactivation by light at a certain wavelength or by photochemical control upon addition of suitable compounds to the imaging solution [41]. These photoswitches can be either organic dyes or fluorescent proteins (FPs). Dickson *et al* showed that fluorescence blinking occurs over time scales of many seconds in single molecules of green fluorescent protein (GFP) upon illumination at 488 nm [42]. The authors also reported that GFP molecules can populate a non-fluorescing dark state from which the photoactive ground state can be repopulated by illumination at 405 nm. Later, improved photoswitches with long lasting non-fluorescent dark states (or OFF-states) were created, e.g. FPs such as PA-GFP [43], Kaede [44], EosFP [45] and Dronpa [46], and organic dyes such as Cy5 or Alexa 647 [47] or the Cy5-Cy3 dye pair [48]. New photo-activatable, -convertible, and -switchable FPs have been created and optimized through site specific mutagenesis of established variants [49–51]. The development of photoswitchable organic dyes is on the other hand driven by a direct understanding of the photophysics associated with the chemical structures of individual dyes [41, 52]. In general, most standard organic dyes can be turned into photoswitches under appropriate buffer conditions [53–61].

In 2006, Betzig *et al* used photoactivatable FPs for super-resolution imaging of cellular structures such as the lysosomal transmembrane protein CD63 in mammalian cells, and termed it photoactivated localization microscopy (PALM) [62]. At the same time, different acronyms for methods based on the same principle have been proposed, such as fluorescence photoactivation localization microscopy (FPALM) [63] with FPs, and stochastic optical reconstruction microscopy (STORM) [64] utilizing a Cy5-Cy3 dye pair. Thenceforth, different refinements have been made such as direct STORM (*d*STORM), a simplified variant of STORM, in which the photoswitching is achieved in single dyes, without requirement for an activator fluorophore [54, 65].

**Figure 2.**
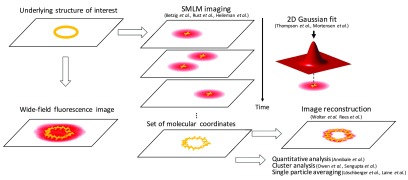
Principle of SMLM. If the underlying structure (here, a yellow circle) is labelled efficiently with appropriate fluorescent dye, it can be imaged with a fluorescent microscope. A conventional wide-field image of this structure will result in a blur limited by the diffraction limit (red blur). However, in SMLM, each fluorophore on the structure of interest is imaged sequentially such that their individual fluorescence pattern can be isolated in each camera frame (indicated by the time axis). The coordinates of each imaged fluorophore can be estimated, typically by 2D Gaussian fit, and the super-resolved image can be computed. Additionally, the set of coordinates can be exploited to perform quantitative analysis, clustering or single particle averaging.

A plethora of super-resolution techniques have since been developed, which exploit the single molecule localization principle, but they are not conceptually different. Variations only exist in the way that fluorescent emitters are made to photoswitch. In all cases, the fluorophores are separated in time and measured as diffraction-limited spots on a 2D array detector (figure 2). The coordinates of each fluorophore are determined with sub-pixel resolution by fitting each emission pattern with a Gaussian function and determining its centre. From a complex sample structure labeled with thousands to millions of fluorophores, only a sparse subset of fluorophores is activated (or switched into a fluorescent ON-state) at any given time in order to permit single-molecule detection and localization. The majority of fluorophores, however, stays in the non-fluorescent OFF-state. Consecutively, the active ON-state molecules are either bleached or switched back to the OFF-state and a new subset of fluorophores is stochastically activated. The sequence is then repeated for many emitter subsets in the sample, and their coordinates are recovered. If enough emitters are localized and the spatial sampling fulfils the Nyquist criterion, the localization coordinates obtained can be used to provide a ‘super-resolved’ representation of the underlying structure. Photoswitching is typically controlled by irradiation with laser light of different wavelengths and/or by chemical buffers. The final resolution of the obtained reconstructed image does not only depend on the precision at which single emitters are localized, but also on the density of labels on the structure of interest and on parameters such as the pixel size of the reconstructed image. Here, the labelling density will define the smallest feature resolvable as described by the Nyquist–Shannon theorem for spatial frequencies in the sample [66, 67].

An alternative approach to obtaining super-resolution images by single-molecule localization in the absence of photoswitching is based on the diffusion and stochastic binding of fluorescent probes onto their target, e.g. cell membranes. The probes become emissive on contact with their target and thus permit their localization. The principle of the technique is summarized in its acronym, PAINT, which is short for ‘points accumulation for imaging in nanoscale topography’ [68]. A variant of the latter technique, uPAINT, was used by Giannone *et al* to obtain super-resolved images and diffusion maps of membrane proteins in living neurons [69]. For a full list of the different SMLM methods and their underlying switching mechanisms, we refer the reader to recent literature [52, 70–72].

## Development of single-molecule localization microscopy

### Image reconstruction

After its first demonstration, SMLM was immediately embraced by many research laboratories around the world. In the early years, the challenges for the technique were mainly of a computational nature, since the efficient localization of millions of single-molecules and the reconstruction of high-resolution images from their coordinates was too time consuming to lead to widespread applicability. Post-acquisition data analysis took hours in the pioneering experiments. However, faster reconstruction methods were soon developed either via improved algorithms [73] or parallelized computing with graphics processing units [74]. Today, reconstruction is not a limitation of the technique and several efficient open source software packages are available to achieve reconstruction on desktop computers within minutes or even seconds [73–78]. For an excellent overview on this topic, we refer the reader to the review of Small and Stahlheber [79].

### 3D imaging

The capability of SMLM has been extended to three dimensions (3D) by ensuring that fluorophores situated at different depths in the sample produce geometrically discernible emission patterns. This can be achieved by engineering the emission PSF through the introduction of astigmatism in the detection path of a wide-field microscope [80]. Variants include the use of a PSF that twists like a double helix along the imaging axis [81], the use of multiple imaging planes [82, 83], and interferometric approaches [84]. Several reviews are available to guide the user towards the most appropriate technique for a given application [85–87].

### Converting quantitative information into molecule numbers

In SMLM, if the emitters are sufficiently sparse, each extracted set of coordinates corresponds to an individual emitter. Therefore, molecular quantification, i.e. counting the number of labeled molecules, is possible. Examples include the counting of subunits in macromolecular complexes labeled with FPs via PALM [88, 89], or, more recently, via *d*STORM [90]. However, artifacts may arise, leading to undercounting, either from imperfect labeling efficiencies or the co-incident blinking of two emitters within the diffraction limit, or overcounting when multiple blinking events from individual fluorophores occur, as previously highlighted [91, 92].

The effects of undercounting and overcounting depend largely on the chosen labelling method, however, none of the labelling methods currently available fully resolve these limitations and, in practice, rigorous control experiments are required.

In theory, genetically-expressed labels such as FPs provide a 1:1 stoichiometry between the number of fluorescent probes and protein molecules of interest but in practice slow, or incomplete, maturation/photoactivation of FPs in cells, can lead to undercounting. On the other hand, when using immuno-labelling in combination with organic dyes, undercounting may be the result of imperfect labeling, which is largely dependent on the affinity and specificity of the antibodies used and the accessibility of the antigen. Other labelling strategies attempted to exploit the 1:1 stoichiometry of genetically-expressed tags with organic dyes, such as Halo [93], SNAP/CLIP [94] or Click chemistry [95] but here again, imperfect labelling efficiencies will result in undercounting. All these effects of undercounting need to be accounted for by estimating the efficiencies of FP maturation, fluorophore photoactivation, and labeling *in situ*.

Overcounting occurs when a single emitter is active for multiple instances during the acquisition sequence. For photoactivatable proteins, such as mEos2, the blinking properties are now well understood. The fluorescence of a single mEos2 molecule occurs typically in rapid sequential bursts of fluorescence intermitted by short dark times before the fluorophore finally bleaches. These dynamics can be modeled and bursts grouped computationally into single events to avoid overcounting [96]. For organic dyes, however, dark times can be very long (e.g. tens of seconds) and thus this grouping approach is not applicable. However, when using indirect immuno-labelling, the localization count per secondary-primary label bound to an epitope can be estimated by taking the average number of localizations per dye-labeled secondary antibody as well as the average number of secondary antibodies per primary antibody [90], thereby making it possible to correct for overcounting.

### Cluster analysis

In many biological systems molecules accumulate in nanoscopic domains to elicit a functional effect; the clustering of receptor proteins on the cell membrane to initiate a signaling pathway is one example. To quantify such assemblies, algorithms that were originally developed in the field of geographical statistics have been adapted to SMLM. They are commonly referred to as cluster analysis. The method has been applied to estimate both size and densities of membrane protein clusters. Notably, the formation of sub-100 nm lipid rafts in the plasma membrane could be verified by cluster analysis and spatial inhomogeneities were quantified using Ripley’s *K* function [97]. Spatial inhomogeneities in protein distributions on the membrane can also be characterized using so called pair-correlation functions, PCF [98] and density based analyses [99]: the latter was used to follow the dynamic clustering of syntaxin at neuronal plasma membranes into nanometer-scale arrangements that orchestrate exocytosis. The method furthermore unraveled the nanometer-scale arrangements of the multi-protein SNARE complexes that underpin neurotransmitter release during synaptic transmission. Cluster approaches using multiple colors were also developed, such as for the observation of HIV virus-cellular host interactions in membranes [100, 101].

### Single-particle averaging

The spatial alignment and averaging of localization data from measurements of multiple structurally identical particles permit a dataset to be obtained with much increased signal-to-noise ratio. Such techniques, termed single particle averaging, are routinely used in high-resolution structural analyses with traditional biophysical tools, such as electron microscopy (EM) based tomography techniques [102]. There is much to be gained to take such approaches into the field of SMLM as has been shown in pioneering recent studies. For example, from particle averaged *d*STORM images the arrangement of gp210 proteins forming the nuclear pore complex could be recovered precisely, and the eightfold symmetry of the complex and the pore formation be resolved with nanometer precision [103]. Other proteins of the nuclear pore complex were subsequently investigated using particle averaging [104], recently even in their 3D arrangements [105].

In a recent example, a model-based fitting approach was used in conjunction with particle averaging to study the precise arrangement of proteins in the Herpes Simplex Virus type-1 (HSV-1) virion with *d*STORM. A precision of ~1 nm was demonstrated with the technique, which has the advantage over EM techniques of a high molecular specificity to discriminate between individual protein species [106]. The study demonstrated furthermore that virus ultrastructure and assembly state can be visualized even inside the cell. In summary, SMLM in combination with particle averaging and model-based analyses using *a priori* information (e.g. EM data on ultrastructure) opens tremendous opportunities for structural biology in the future.

These examples make clear, that the future of SMLM depends critically not only on further progress in dye technology and labels, but also on the development of sophisticated reconstruction and analysis algorithms [106–108].

## Applications in the field of neurosciences

Although super-resolution techniques such as STED [109] are significantly contributing to research in the neurosciences [110–112], we focus here on SMLM imaging in this application section. We begin with examples from general neurobiology and conclude with examples of research into neurodegenerative diseases.

### General neurobiology

**Figure 3.**
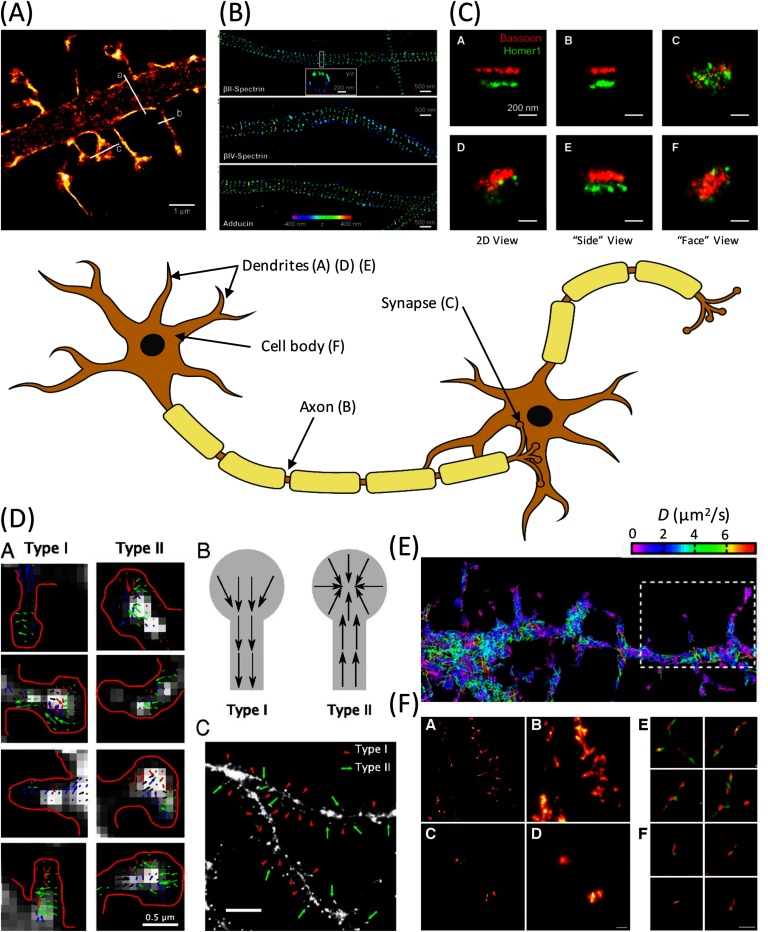
Single molecule localization microscopy, SMLM, uncovers the molecular anatomy of the neuron. The cartoon of two connecting neurons in the centre panel illustrates major components of the neuronal architecture, from which information has been gained with super-resolution microscopy. Labels in parentheses refer to the approximate locations for which example data from SMLM are shown in panels (A)–(F). Panels: (A) Study of actin dynamics in living dendritic spines. The changes in spine morphology was investigated using time-lapse PALM (reproduced with permission from [116]). (B) Observation of the periodic structure of actin, spectrin and adducin in axons. The images were obtained using multi-color 3D STORM (reproduced with permission from [2] copyright 2013 AAAS). (C) 3D STORM imaging in synapses reveal the distribution of Bassoon and Homer1 proteins and thus permit, respectively, a clear differentiation of the pre- and post-synaptic termini (reproduced with permission from [119]; copyright 2010 Elsevier). (D) Observation of AMPA receptor trafficking at the single molecule level. Here, the authors use single-particle tracking PALM (sptPALM) to extract local velocity maps of AMPA receptors transported in living dendritic spines: 2 types of spines are observed (reproduced with permission from [121]; copyright 2012 National Academy of Sciences). (E) Diffusion maps for the photoactivatable, lipophilic dye DiI on dendritic membranes obtained by single-particle tracking (reproduced with permission from [122]; copyright 2012 National Academy of Sciences). (F) *In vitro* assay to probe the seeding activity of amyloid-*β* from patient derived cerebrospinal fluid with amyloid-*β*_1–40_ peptide via 2 color *d*STORM (reproduced with permission from [143]; copyright 2014 Oxford University Press).

The potential of SMLM for studies in the neurosciences was embraced from the early days of the technique and groundbreaking discoveries have been made since. In figure 3 a range of image panels are shown that depict examples where SMLM experiments have shed new light on the architecture and function of neuronal components. The cartoon of two interconnected cells in the middle is included to provide a context for the examples shown and is labelled to provide positional context for panels (A)–(F).

Key questions related to memory and learning concern how neuronal activity changes the shape and morphology of synaptic connections, a topic generally referred to as synaptic plasticity [113, 114]. Synapses can strengthen and weaken as neuronal activity increases or decreases and these changes are in turn caused by dynamic changes in the actin cytoskeleton. PALM was successfully applied for studying actin within living spines [115, 116]. For instance, Izzedin *et al* were able to perform high resolution SMLM imaging of dendritic synaptic spines (see figure 3, panel (A)) using the actin-binding, photoactivatable fusion protein construct ABP-tdEosFP [116]. Impressively, the authors were able to perform long term dynamic PALM imaging in live neuron cultures to follow changes in spine morphology and underlying cytoskeleton structure.

The transmission of signals from the cell body of a neuron to an axonal synapse occurs via changes in action potential along the axon and is mediated by sodium channels distributed across the axonal membrane. Dramatic new insights into axonal structure were gained by 3D STORM imaging of the cytoskeletal proteins actin, spectrin, and adducin (figure 3, panel (B)). Intriguingly, and in stark contrast to dendritic structures, where linear actin filaments run primarily along the dendrite axis, actin filaments in axons were found to be arranged in periodically spaced rings, spanning the circumference of the axonal tube [2]. The distance between the actin rings was found to be consistent with the size of spectrin tetramers, which permitted the authors to suggest a new structural model to underpin sodium channel arrangements in axons. In a different context, two-color *d*STORM was more recently applied to observe structural arrangements of proteins in axonal mitochondria, revealing that the protein UCP4 may exclusively act as a reactive oxygen species regulator [117].

The transmission of signals between neurons is mediated via the synaptic cleft, formed between the transmitting neuron and the receiving neuron. Learning and memory are likely encoded in the number and type of synaptic connections formed in neuronal tissue [118]. Distinguishing the pre- and post-synaptic termini and obtaining data on the morphology of, and protein distributions near, the synaptic cleft have been formidable challenges because of the small size of features involved. Two color SMLM imaging has overcome this problem. In panel (C), figure 3, taken from Dani *et al* [119], the distributions of the pre- and post-synaptic proteins Bassoon and Homer1 are clearly differentiated across the synaptic clefts of neurons in brain tissue. This and related studies show that the precise structural arrangement of synaptic proteins can now be quantified by SMLM, revealing extraordinary new details on neurotransmitter receptor organization and activity dependent plasticity [90, 120].

The study of the dynamic trafficking of receptor proteins and their assembly and disassembly into functional clusters is powerfully enabled by single particle tracking approaches. Hoze *et al* combined the PALM approach with single-particle tracking (sptPALM) and measured local velocity and diffusion coefficients of AMPA receptors, which regulate excitatory postsynaptic potentials, in individual dendritic spines (see panel (D), figure 3) [121]. Using advanced particle tracking algorithms, the authors were able to identify two classes of spinal protrusions, which featured either inward or outward trafficking of AMPA receptors, respectively. Similarly, Shim *et al* studied the diffusion of the lipophilic, photoswitchable dye DiI on the membrane of dendritic spines, and generated diffusion maps in this way [122]. They were thus able to correlate changes in local diffusion coefficients with the underlying dynamics of the membrane ultrastructure (figure 3, panel (E)).

### Neurodegeneration

As a final example of this review we focus on the use of SMLM techniques to provide new insights on mechanisms causing the degeneration of neurons upon aggregation of disease-related proteins. Neurodegenerative diseases, such as Alzheimer’s, Parkinson’s and Huntington’s, are all characterized by the misfolding of protein species and their consequential aggregation within the brain of patients, resulting in neuronal death and loss of cognitive function. The peptides involved differ greatly between the different types of disease (*α*-synuclein for Parkinson’s, Tau and A*β* for Alzheimer’s, and polyQ-Huntingtin in the case of Huntington’s), yet in their aggregated forms, these proteins feature remarkable structural similarities, adopting beta sheet rich fibrillar structures, so called amyloids [123, 124]. Traditional, non-optical techniques, such as atomic force microscopy (AFM) or EM, have provided structural details on individual aggregate and revealed polymorphisms, i.e. they are capable of differentiating between oligomeric or fibrillar protein forms. However, these methods do not permit *in situ* observations of amyloid localization and formation.

A key question in the context of disease is to establish which polymorphic species elicit a toxic cellular response [125]. To address such questions, the development of *in situ* and *in cellulo* assays is essential. Optical techniques permit protein aggregation to be studied *in vivo* and to put the observations into context with disease related phenotypes [126]. However, in the past, the techniques relied on indirect readouts of aggregation state [126–129]. The resolution of SMLM techniques has now advanced to a stage where the direct imaging of amyloid morphology is possible *in vitro* at single molecule resolution. The method was originally demonstrated using blink microscopy of polyQ aggregates [130] and later followed by the first *in situ* observation of A*β* aggregates in cells via *d*STORM [131]. The latter study highlighted the influence of the cellular environment on aggregation kinetics and morphologies, differing markedly from what was observed for comparable conditions *in vitro*, in the test tube. In contrast, fibrillar polyQ species appeared to be more similar, structurally, between the corresponding *in vitro* and *in cellulo* cases [132]. Similarly, the aggregation of human lysozyme, which occurs in a certain case of hereditary amyloidosis linked to specific lysozyme mutations, were observed both *in vitro* and in cells using *d*STORM and showed similar morphological features [133].

These pioneering studies have paved the way for more detailed biophysical and biological studies. Notably, the growth (polymerisation) of amyloid species can now be monitored *in situ* using multi-color SMLM approaches and new insights into the nature of amyloid elongation rates were obtained for *α*-synuclein [134, 135] and polyQ [136–138]. Whilst the former studies were performed *in vitro*, in test tube samples, similar investigations can now be performed directly in cells. Thus it was possible to reveal mechanisms of Tau protein propagation from cell to cell and a prion like proliferation of aggregating species was observed with two color SMLM, which shed new mechanistic insights into Alzheimer’s disease (AD) [139]. The latter finding highlighted the potential risk associated with increased levels of extracellular Tau, as may result, for example, as result of neuronal death associated with repeated trauma to the head region, providing a possible link to sports injury related dementia. A similar ‘prion like propagation’ mechanism was recently verified for *α*-synuclein, using similar methods [140]. In another study, SMLM revealed that the aggregation kinetics of mutant variants of A*β* are faster in cells than wild type A*β*, again providing a link to AD disease pathology [141, 142]. This provides a direct connection to a more recent study using samples from AD patients: Using two-color SMLM it was observed that A*β* obtained from the soluble fraction of brain extracts of AD patients have a higher capacity to propagate the amyloid state than that extracted from cerebrospinal fluid [143] (figure 3, panel (F)). The experiments reviewed here were obtained with immunofluorescence or covalently labelled amyloids, however, other labelling approaches have also been successfully combined with SMLM, such as using intercalating dyes that are specific to amyloids [144]. SMLM is clearly making a tremendous impact in the study of protein misfolding diseases, *in situ*, along with other molecular probing techniques such as FRET [127, 145]. However, the field is still young and numerous questions remain to be answered, such as, what is the interaction of each amyloid species with the cellular machinery? Which species are responsible for cell death? What is the effect of the presence of these fibrillar structures on synaptic transmission? Super-resolution fluorescence techniques will provide many answers to these related questions, lifting the cover off the molecular phenomena at the root of these pathologies and may pave the way for future therapeutic strategies.

## Conclusion

The immense scientific progress made over the last 25 years, both from technological and theoretical points of view, have caused a revolution in the field of optical microscopy. Modern variants enable scientists to observe molecular species directly in their native environments and in a context relevant to biomedical research. In the neurosciences in particular, the impact of super-resolution imaging is spectacular. Imaging techniques will be the key to unlock the mysteries underlying the function of the brain, which remains as one of the greatest scientific challenges to mankind, as highlighted by the launch of the BRAIN initiative. SMLM and other super-resolution techniques have begun to unravel key phenomena providing new insights into the micro-physiology of the brain and molecular events at the focus of neurodegenerative diseases such as Alzheimer’s, Parkinson’s or Huntington’s diseases.

In the future, we expect that combinations of super-resolution techniques with non-optical techniques such as correlative optical/AFM [146] or optical/EM [147, 148] or combination of structural optical tools with functional imaging such as Fluorescence lifetime imaging (FLIM) and Förster resonance energy transfer (FRET) will provide even deeper insights into the intricate links between molecular structure and function and pathological phenotypes.
